# Simultaneous Surface-Near and Solution Fluorescence Correlation Spectroscopy

**DOI:** 10.1007/s10895-016-1789-0

**Published:** 2016-03-22

**Authors:** Christian M. Winterflood, Stefan Seeger

**Affiliations:** Randall Division of Cell and Molecular Biophysics, King’s College London, London, SE1 1UL UK; Department of Chemistry, University of Zurich, Winterthurerstrasse 190, 8057 Zurich, Switzerland

**Keywords:** Fluorescence correlation spectroscopy, Supercritical angle fluorescence, Undercritical angle fluorescence, Surface-selective, Near-field, Far-field

## Abstract

We report the first simultaneous measurement of surface-confined and solution fluorescence correlation spectroscopy (FCS). We use an optical configuration for tightly focused excitation and separate detection of light emitted below (undercritical angle fluorescence, UAF) and above (supercritical angle fluorescence, SAF) the critical angle of total internal reflection of the coverslip/sample interface. This creates two laterally coincident detection volumes which differ in their axial extent. While detection of far-field UAF emission producesa standard confocal volume, near-field-mediated SAF produces a highly surface-confined detection volume at the coverslip/sample interface which extends only ~200 nm into the sample. A characterization of the two detection volumes by FCS of free diffusion is presented and compared with analytical models and simulations. The presented FCS technique allows to determine bulk solution concentrations and surface-near concentrations at the same time.

## Introduction

For the study of processes at surfaces and interfaces the standard confocal FCS has the immanent problem that the ellipsoidal observation volume suffers from having a low axial confinement. As a result, surface processes remain concealed by the background produced by the bulk fluorescence.

Optical near fields have been succefully used to confine observation volumes to interfaces. FCS has, for instance, been performed using evanescent waves produced at optical nanostructures called zero-mode waveguides [1–3] or more commonly using TIRF [4–7]. TIR-FCS uses objective-type TIRF illumination to restrict the excitation to a thin section less than 200 nm above the interface in combination with standard confocal detection to ensurse the lateral confinement of the detection volume. TIR-FCS has proven very useful for the study of processes close to a surface/solution interface. In theory, it can give access to a number of properties, including local fluorophore concentrations and local fluorophore translational mobility [8], or kinetic rate constants for reversible association of fluorophores with the interface [9]. The determination of these quantities by TIR-FCS, however, relies on the a priori knowledge of the fluorescent solution concentration. In many biological cases, though, such as the study of the interaction of proteins with membranes or membrane proteins, rely on the use of fluorescent fusion proteins whose cellular expression levels are not precisely known [10]. While the advantages of SAF-CS have already been described [11], in this report we provide an extension to the technique which allows to perform FCS in close proximity to the sample/solution interface as well as deeper in solution simultaneously.

## Results

**Fig. 1:**
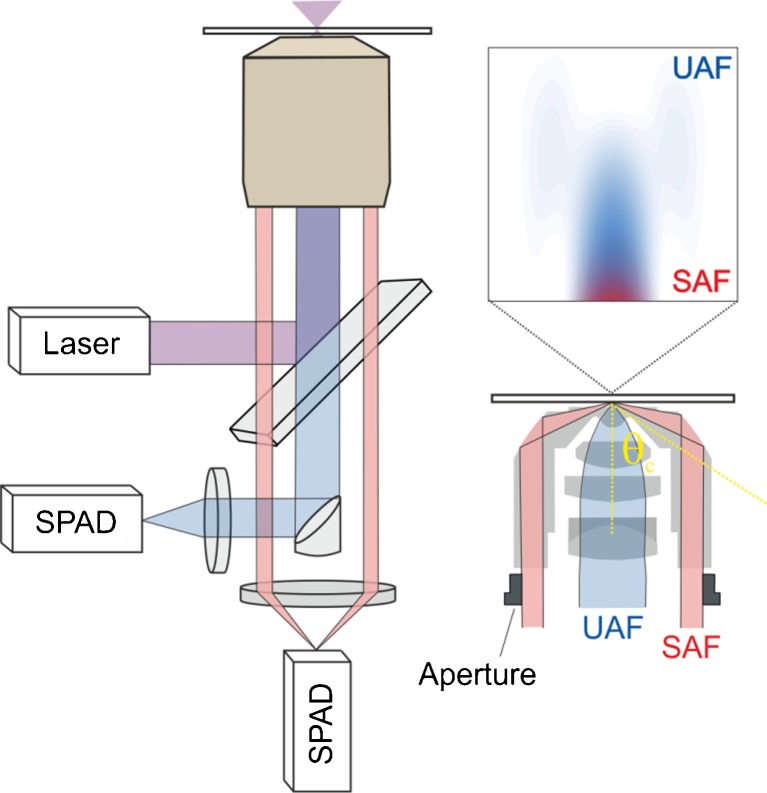
Schematic of the optical setup

We use make use of a previously reported custom setup and microscope objective [12] (Fig. 1) for tighly focused, undercritical angle excitation and parallel, well-separated collection of SAF and UAF. SAF collection yields a highly surface-confined detection volume, while UAF collection yields a conventional confocal volume which extends deeper into the sample. The simultaneous measurement of SAF and UAF has been used for determining axial emitter positions with nanometer accuracy [13] as well as to reduce artifacts in membrane FCS related to a non-planar geometry of the membrane [14].

Quantitive results in FCS rely on the size and shape of the detection volume. The most common way of calibrating the detection volume is to perform FCS on a fluorescent species with known diffusion coefficient and concentration. While the temporal decay of the autocorrelation function (ACF) depends on the shape of the observation volume, the amplitude of the ACF gives direct access to the size of the detection volume through the relationship *V*_eff_ = 1/(*G*_0_×*C*). Here, *V*_eff_ is the socalled effective volume, *G*_0_ the amplitude of the ACF, and *C* the concentration of the sample. In turn, it is possible to determine concentrations of fluorescent species with a calibrated effective volume. We carried out diffusion measurements on the red fluorescent dye Atto655 (in its carboxylic acid form, −COOH) which has negligible triplet state contributions and a precisely determined diffusion coefficient [15]. A difficulty when trying to probe the detection volume at the coverslip/solute interface by free diffusion arises from non-specific interaction of the fluorophore with the coverslip glass. This flaws the ACF in that it is shifted to longer decay times while the amplitude is decreased. Accordingly, great care needs to be taken for the preparation of the coverslip.

**Fig. 2:**
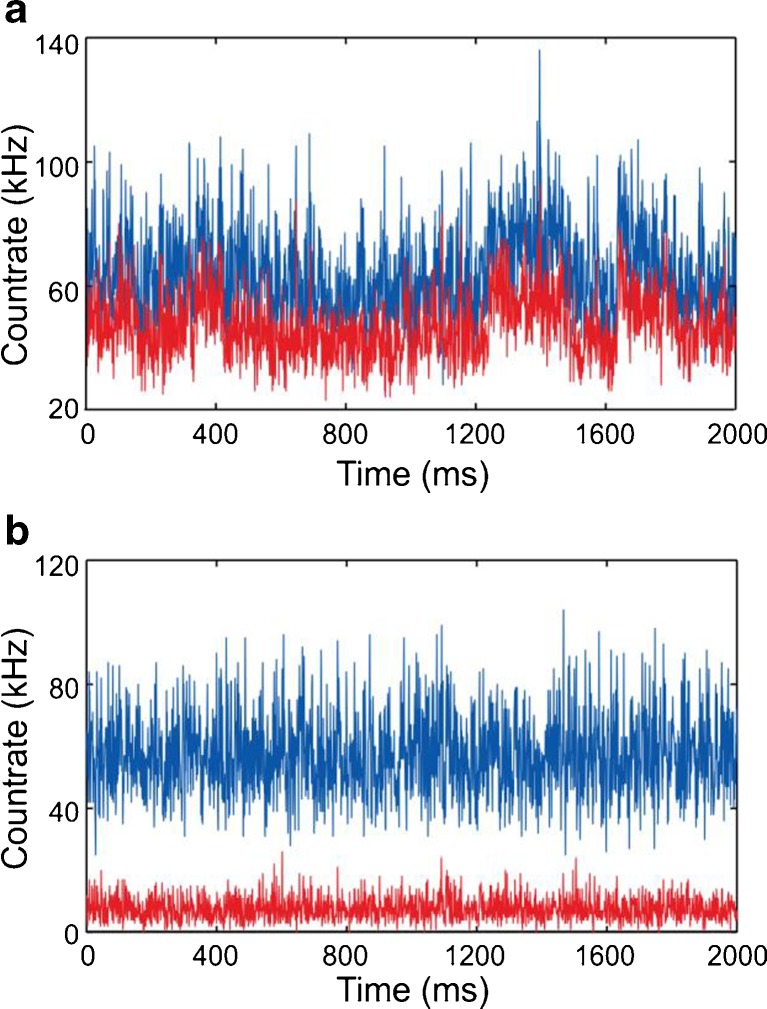
SAF (*red*) and UAF (*blue*) intensity tracks with (**a**) and without non-specific adsorption (**b**) to the coverslip glass. The sample was 10 nM Atto655 in 200 mM NaCl. The excitation intensity was 13 μW

Figure 2 (*bottom graph*) shows the parallel detection of SAF and UAF of a 10 nM solution of Atto655 with a plasma-treated coverslip and at high ionic strength (200 mM NaCl) to shield the electrostatic repulsion between the negatively charged dye (net charge of −1) and the glass [16]. In comparison, Fig. 2 (*top graph*) shows the intensity tracks of SAF and UAF using a non-plasma treated coverslip with pronounced non-specific adsorption. A 63° cut-off was used for SAF (critical angle for water/glass: 61.9°).

**Fig. 3:**
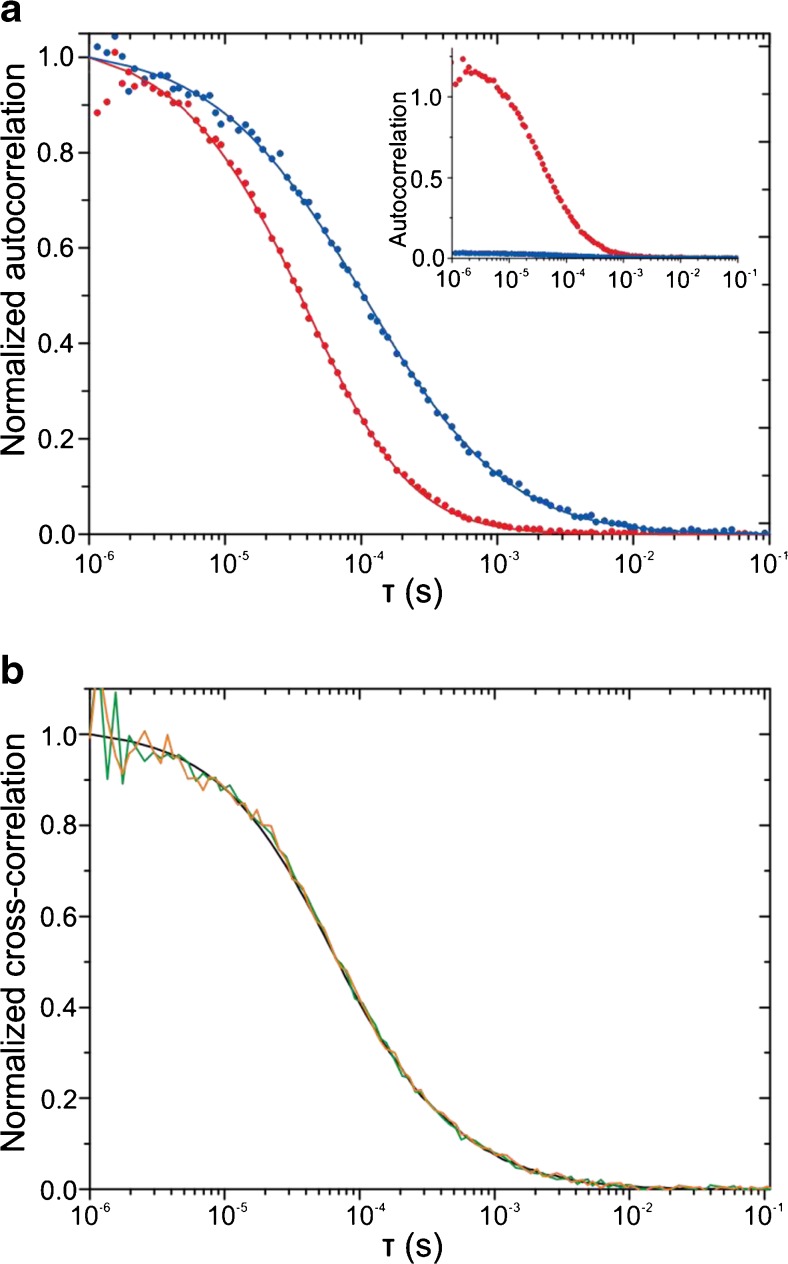
**a** Afterpulsing corrected SAF (*red points*) and UAF (*blue points*) ACFs for free diffusion of a 10 nM solution of Atto655 fitted with their respective models (*lines*) and the corresponding non-normalized ACFs (*inset*). **b** Simulated (*black line*) and experimental cross-correlation functions SAF ⋆ UAF (*green line*) and UAF ⋆ SAF (*magenta line*) for freely diffusing Atto655. Laser power 13 μW, acquisition time 200 s

Figure 3a shows the parallel FCS measurement with SAF and UAF of freely diffusion Atto655. The amplitude (*G*_0_) of the ACF for SAF was over thirty times larger than for UAF, given the substantially larger detection volume (Fig. 2, inset). The UAF ACF was fitted to the standard three-dimensional Gaussian model (Eq. 1 from Ref. [11]) while the SAF ACF was evaluated according to Eq. 5 from Ref. [11]. The average of six separate FCS measurements, each with different lateral positions on the coverslip and newly adjusted focus, gave an effective volume *V*_*eff*_ = 144.0 ± 1.3 aL for SAF and *V*_eff_ = 5.49 ± 0.07 fL for UAF. Notably, the relative error for both the SAF and UAF effective volumes is around 1 %. Theoretical values for *V*_eff_ were calculated directly from the observation volume spatial profile according to Eq. 23 in Ref. [17] and gave *V*_eff_ = 136.7 aL for SAF and *V*_eff_ = 6.50 fL for UAF, which is in good agreement with experimentally determined values.

The comparatively large effective volume for UAF is because we used the larger photosenstive area of the detector of 180 μm diameter as a pinhole (corresponding to 4.5 Airy units). This was to ensure that the excited area at the coverslip/sample interface coincided with both detection volumes to ensure that both SAF and UAF detection volumes were interrogating the same area.

With SAF and UAF being measured in parallel we additionally evaluated the cross-correlation functions SAF⋆UAF and UAF⋆SAF (Fig. 3b). We compared the experimental cross-correlation functions with simulations and there was a good agreement. Although a model for the cross-correlation of SAF and UAF currently lacks, it is conceivable that it contains information on directional transport along the *z*-axis or irreversible binding processes.

**Fig. 4:**
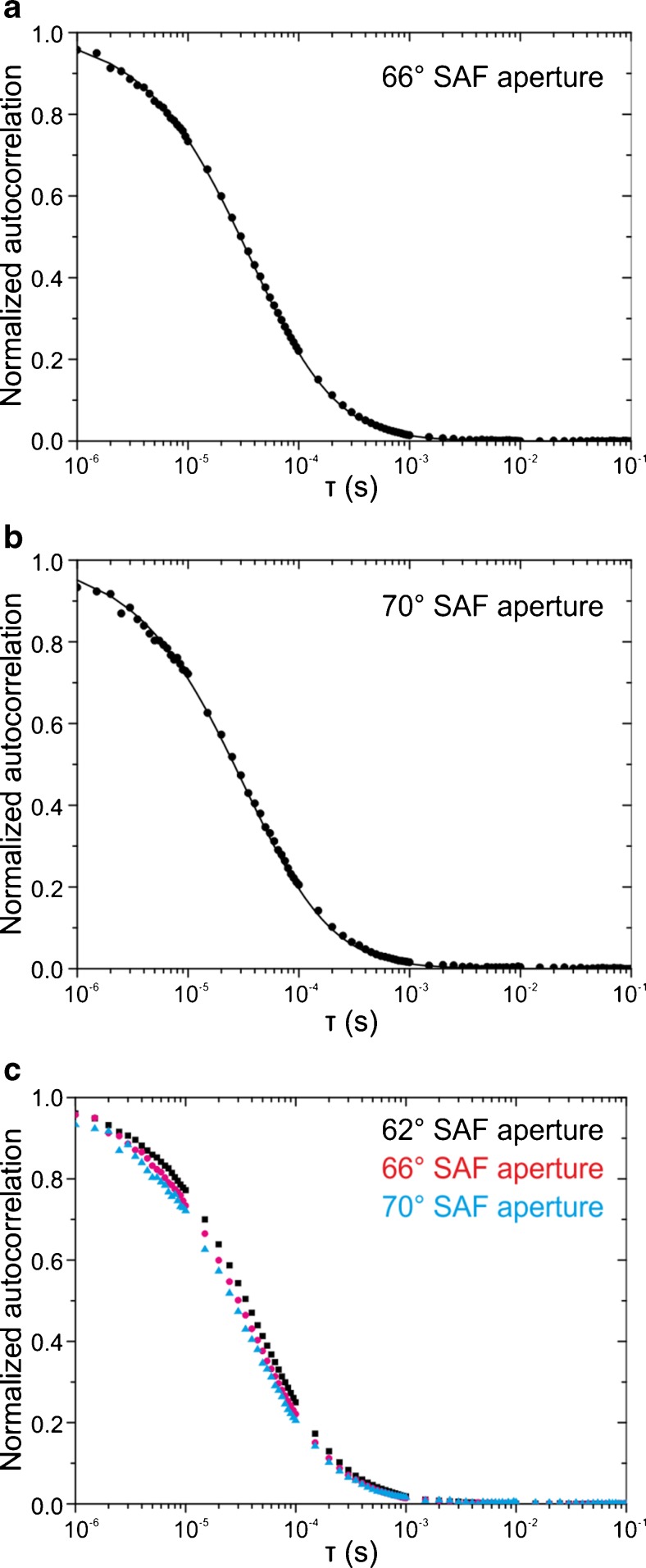
Afterpulsing corrected SAF ACFs for different SAF cut-off angles of a 10 nM solution of Atto655 fitted with their respective models (*solid lines*). Laser power 13 μW, acquisition time 200 s

It is possible to further axially confine the detection for SAF by increasing the cut-off angle of fluorescence collection. For this we show FCS measurements of freely diffusing Atto655 carried out with higher SAF cut-off angles. Experimental SAF ACFs for cut-off angles of 66° and 70° are shown in Fig. 4.

The experimentally determined effective volumes for SAF were *V*_eff_ = 114.5 ± 1.0 aL (theory: 112.2 aL) for the 66° and *V*_eff_ = 127.7 ± 4.2 aL (theory: 98.1 aL) for the 70° SAF-aperture. While the decays of the SAF ACFs are good agreement with the analytical model, the experimental value for the 70° aperture is significantly larger than the theoretical value–even larger than compared to the 66° aperture. However, fluorescence collection this far above the fluorescence maximum at the critical angle comes at a larger loss of fluorescence signal and statistical accurracy and is therefore less practicable. For freely diffusing Atto655 a countrate per molecule *cpm* of 54.4 kHz for SAF and 28.5 kHz for UAF was calculated for a measurement using 67 μW excitation intensity. This corresponds to a molecule brightness *mB* of 8.2 × 10^5^ W^−1^ and 4.3 × 10^5^ W^−1^ for SAF and UAF, respectively.

In summary, the first simultaneous measurement of surface-near and solution FCS was described and a detailed quantification of the custom optics by FCS was provided. It is noteworthy that the method is not restricted to our specialized optics. It could in principle be performed with conventional high NA objectives as the separate detection of SAF and UAF has already been demonstrated [18]. Our approach can be used for measuring weak or transient interactions at surfaces or membranes with unknown solution concentrations by FCS.
